# Understanding the spatial distribution and hot spots of collared Bornean elephants in a multi-use landscape

**DOI:** 10.1038/s41598-022-16630-4

**Published:** 2022-07-27

**Authors:** N. K. Abram, B. Skara, N. Othman, M. Ancrenaz, K. Mengersen, B. Goossens

**Affiliations:** 1 Forever Sabah, H30 Gaya Park, Lorong Muntahan 1C, Penampang Road, 88300 Kota Kinabalu, Sabah Malaysia; 2 Institute for Biodiversity and Ecosystem Dynamics, University of Amsterdam, Science Park 904, 1098 XH Amsterdam, The Netherlands; 3 Sabah Biodiversity Conservation Association (Seratu Aatai), S10, 1st Floor, Block B, Peak Vista, 88400 Kota Kinabalu, Sabah Malaysia; 4 Institute for Tropical Biology and Conservation, Universiti Malaysia Sabah, 88400 Kota Kinabalu, Sabah Malaysia; 5 HUTAN/Kinabatangan Orang-Utan Conservation Programme, 88874 Kota Kinabalu, Sabah Malaysia; 6 Centre for Data Science and School of Mathematical Sciences, Queensland University of Technology, 2 George St, Brisbane, QLD 4001 Australia; 7 Danau Girang Field Centre, c/o Sabah Wildlife Department, Wisma Muis, 88100 Kota Kinabalu, Sabah Malaysia; 8 Organisms and Environment Division, Cardiff School of Biosciences, Cardiff University, Sir Martin Evans Building, Museum Avenue, Cardiff, CF10 3AX UK; 9 Sustainable Places Research Institute, Cardiff University, 33 Park Place, Cardiff, CF10 3BA UK; 10 Sabah Wildlife Department, Wisma Muis, 88100 Kota Kinabalu, Sabah Malaysia

**Keywords:** Ecology, Zoology, Environmental sciences

## Abstract

In the Kinabatangan floodplain, Sabah, Malaysian Borneo, oil palm and settlements have reduced and fragmented lowland tropical forests, home to around 200 endangered Bornean elephants (*Elephas maximus borneensis*). In this region, elephants range within forests, oil palm and community areas. The degree to which elephants are using these areas remains unclear. We used GPS telemetry data from 2010 to 2020 for 14 collared elephants to map their entire known ranges and highly used areas (hot spots) across four land use categories and estimate time spent within these. The use of land use types across elephants varied significantly. Typically, females had strong fidelity to forests, yet many of these forests are threatened with conversion. For the three males, and several females, they heavily used oil palm estates, and this may be due to decreased landscape permeability or foraging opportunities. At the pooled level, the entire range and hot spot extents, constituted 37% and 34% for protected areas, respectively, 8% and 11% for unprotected forests, 53% and 51% for oil palm estates, and 2% for community areas. Protecting all forested habitats and effectively managing areas outside of protected areas is necessary for the long-term survival of this population.

## Introduction

Increasing human populations and greater demands for certain commodities have resulted in large-scale conversion of natural ecosystems to agriculture and other human land uses^1,2^. Such habitat loss is having significant negative impacts on biodiversity globally, with rates predicted to increase in tropical biomes and biological hot spots^3,4^. Deforestation and habitat conversion are major threats to large ranging species like elephants as they are increasingly left within landscapes with little and fragmented remaining natural habitat, often not large enough to sustain viable breeding populations^5^. Moreover, poorly planned land use changes and aggressive profit driven management of landscapes often result in a lack of habitat connectivity for elephants^6^. Elephants are increasingly roaming outside of the network of protected areas, and there is an urgent need to understand how they use these multiple-use landscapes to improve conservation and effective wildlife management^7^.

The Bornean elephant (*Elephas maximus borneensis*) is a prime example of a species that is threatened by habitat conversion of natural forest to agriculture and other types of man-made landscapes^7–9^. Bornean elephants are an endemic sub-species whose range is limited to approximately 5% of the island of Borneo, occurring largely in the central and south-eastern region of the Malaysian State of Sabah^10–13^. In Sabah, elephants are found within three major elephant managed ranges, the Lower Kinabatangan, Central Sabah and Tabin^14^. These ranges are isolated from each other due to large tracts of agricultural landscapes, linear infrastructures (roads) and human settlements^14,15^. In fact, Sabah has lost over 40% of its forest over the last 30 years, most of it being replaced with oil palm monoculture in the east and south-eastern lowlands^1,16^. Over the past 15 years or so, elephants have been increasingly observed within human landscapes, including oil palm estates and smallholdings, human settlements, and along linear infrastructures (roads), heightening human–elephant encounters and conflicts^15^. However, the frequency and extent to which elephants enter human-dominated landscapes have not been quantified yet. Around 200–250 elephants are currently found in the Lower Kinabatangan^14^. This population has the smallest range in Sabah, confined between the mangrove forests to the east and the Batu Putih Road Bridge to the west, which is located half-way up the Kinabatangan River and has proven to be an impassable barrier for elephant movements since the early 2000’s^14^.

Elephant spatial distributions are a reflection of ranging behaviour^17^, social organization^18^ and ecological needs^19–23^. Family groups show strong fidelity to their home ranges, which commonly include distinct seasonal ranges^24,25^. However, extreme droughts and human disturbances^18,26,27^, overpopulation^8,28^, habitat degradation and/or transformation^29^, and other factors such as major linear infrastructure developments (e.g. roads and rail)^30,31^, are all factors that affect home ranges and seasonal migratory patterns of elephants. In fact, elephants are highly adaptable and can modify their behaviour, to some degree, to cope and adapt to habitat changes. For example, males, especially those that have formed long-term, stable all male groups, sometimes use potentially “risky” areas such as agricultural landscapes and settlements^32–34^. Females, on the other hand, are typically less adverse to risky areas that they tend to avoid; they have also been found to reduce vocalizations and stay in tighter groups whilst outside of forested areas to minimise detection^35^.

Habitat suitability models are commonly applied to determine the location of wildlife corridors or linkages in human-dominated landscapes^36,37^. However, where a species utilizes landscapes dominated by monoculture crops with minimal heterogeneity, such as oil palm, model outputs may not produce meaningful results. Another approach is to apply hot spot analysis within a species geographic range (i.e., the extent of occurrence) to map the frequency or weight of where a species is occurring across a landscape^38–41^. A hot spot is defined here as an area with statistically significant spatial clusters of either high or low values^42^. Hot spot analyses are useful in identifying important ecological biogeography patterns and habitat features, as well as anthropogenic characteristics associated with species distributions^38–41^.

In this study, we identify the distribution (entire known range) and hot spots of 14 GPS collared Bornean elephants (11 females and three males) living in the Lower Kinabatangan, from 2010 to 2020, and consider these at the pooled, clustered, and individual levels. We develop spatial land use and land cover data for 2010 and 2015 to understand the extent and proportion of time spent within differing land use categories within the elephant’s hot spots and compare this with their known ranges. We also look at hot spots on, or near to, a planned highway alignment to understand the potential impact of this infrastructure development project. Lastly, we look at time spent by elephants in different oil palm estates to identify where better management strategies are needed to improve habitat permeability and reduce human–elephant conflicts^40,43,44^.

## Methods

### Study area

**Figure 1:**
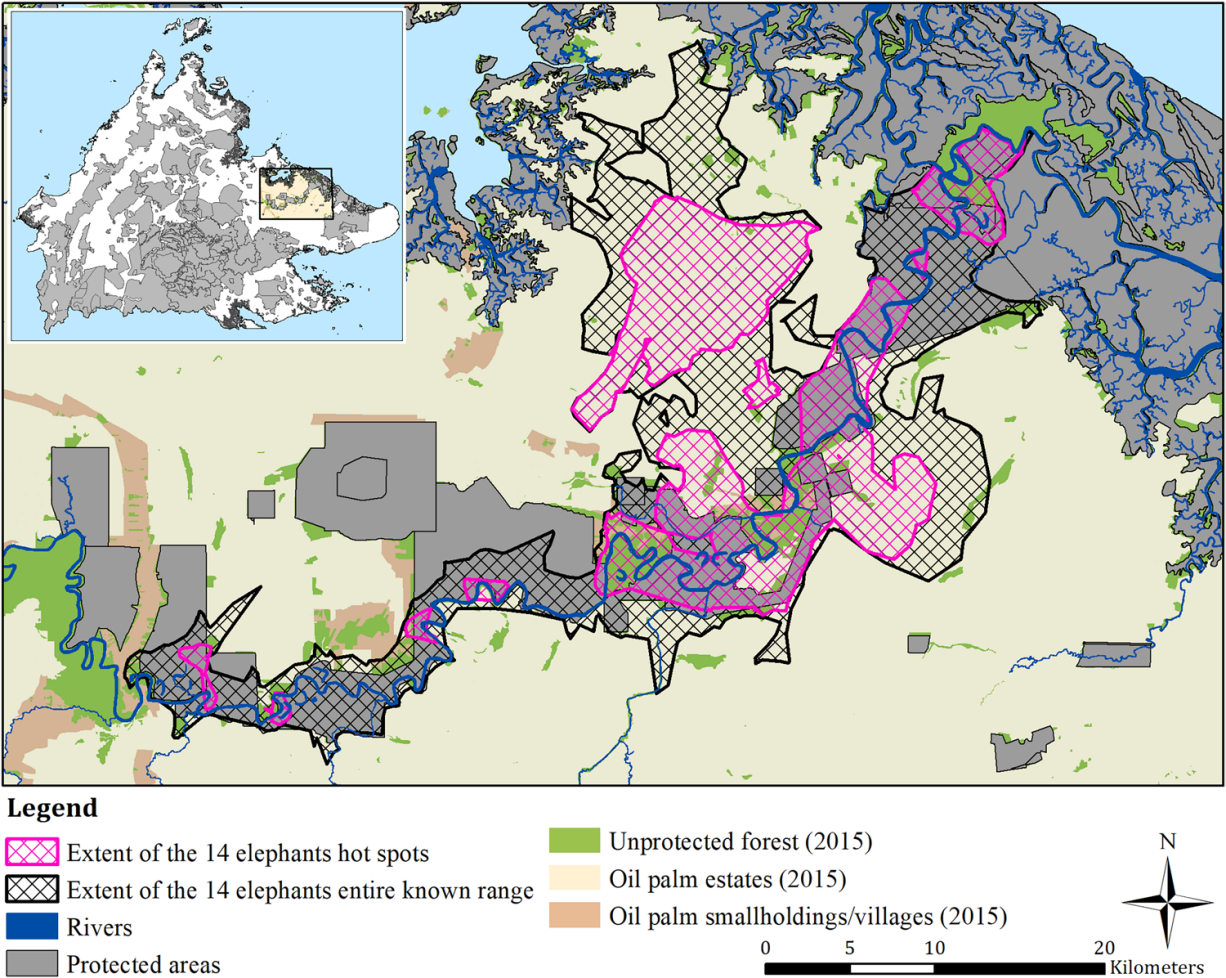
Map showing the pooled extents for all 14 elephant individuals for their collective hot spot areas (pink cross hatch) inside of their collective entire range (black stripped area) within the Lower Kinabatangan in eastern Sabah.

**Figure 2:**
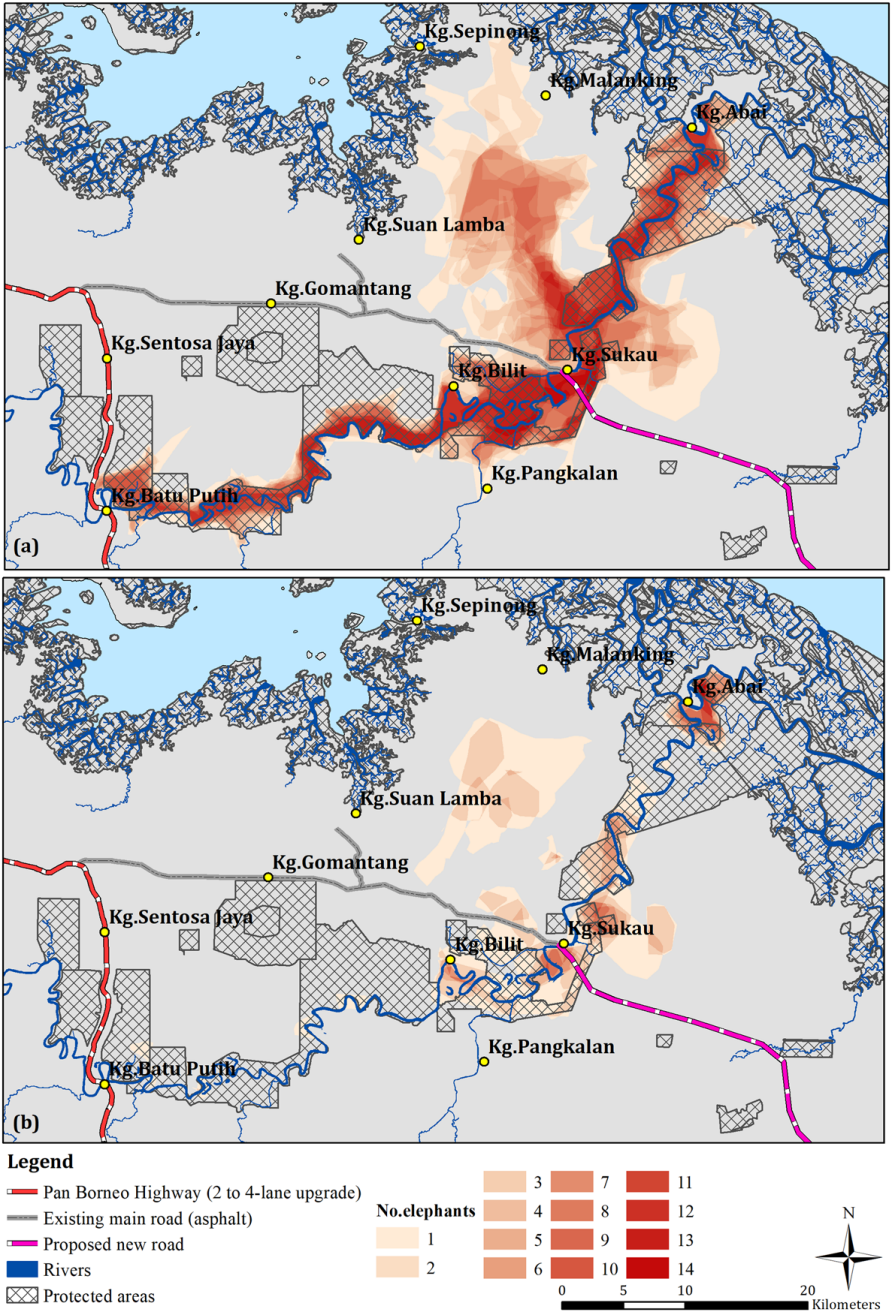
Heat maps showing the cumulative areas of elephant ranges for their entire range (**a**) and hot spot areas (**b**) along with information on the planned 4-lane Pan Borneo Highway from the current existing 2-lane road (red), other planned new road (pink) and the current existing road (grey), along with Protected Areas/Forest Reserves (black cross hatch).

We restricted the study area to the general geographical extent of the Kinabatangan elephant population in eastern Sabah, a Malaysian state located in north Borneo (Fig. 1). The Kinabatangan elephant population’s range encompasses areas north and south of the Kinabatangan River, the largest river in Sabah, and extends from Batu Putih village in the west to Abai village in the east (Fig. 2)^9,45^. Large-scale timber extraction, in this region, started in the late 1950s, followed by intense conversion of logged forests to large-scale oil palm estates from the 1980s onwards^46^. Today, the landscape is dominated with oil palm that is perforated with a number of small, fragmented protected areas (Wildlife Sanctuary and Virgin Forest Reserves), as well as unprotected forest fragments, roads, and human settlements^47^. The villages within this region, are largely dependent on income from their oil palm smallholdings as well as from wildlife-watching tourism. The region experiences mean monthly temperatures of 21–34 °C and average annual rainfall of 3000 mm^48^. Due to the high annual rainfall, the low lying floodplain experiences significant seasonal flooding events, which has been documented to effect elephant range areas^9^.

### Study species

The Bornean elephant is an endangered sub-species of the Asian elephant (*Elephas maximus*), listed in Schedule 1 (Totally Protected Species) of the Sabah Wildlife Conservation Enactment 1997, with a total population size estimated to be below 2000 individuals^7,14^. As a result of restricted distribution ranges^10^, habitat fragmentation^9^, and a lack of linkages within the landscape^49^, current Bornean elephant populations are subjected to high levels of inbreeding^7^. In the Lower Kinabatangan floodplain, the elephant population size is estimated between 200 and 250 individuals, with population density estimates of 2.15 elephants/km^2^^2,50^.

### Collaring of elephants, ethical approval and informed consent

Selection criteria for collaring individuals was based on sex and rank in the family unit, and from knowledge of the field team who follow the elephants on a regular basis. The study is reported in accordance with ARRIVE guidelines. The anesthesia of elephants during collaring was carried out by a certified veterinarian from the Sabah Wildlife Department. All efforts were made to reduce stress and ensure the welfare of the animals. Capture and handling protocols were approved by Sabah Biodiversity Centre (permit JKM/MBS.1000-2/2 JLD.4) and carried out in accordance with the current laws of Malaysia and Sabah Wildlife Department’s Standard Operation Procedures on Animal Capture, Anaesthesia and Welfare. Animals were anaesthetised by a sedative agent (Xylazil-100) administered by a wildlife veterinarian. Once sedated, the elephant was kept in a standing position by using small poles pointed at the back of its ears and the collar with the tracking device was attached around its neck. To minimize unnecessary time sedated, we used a reversal administered intra-veinously (Yohimbine) as soon as the collaring was completed.

### Mapping elephant ranges and elephant hot spot areas from collared GPS data

#### Mapping elephants entire ranges

Satellite Global Positioning Systems collars (IR SAT (GPS/UHF), iridium, 12 D cells) from Africa Wildlife Tracking™ (AWT, Pretoria, South Africa) were fitted to 14 adult individuals (11 females and three males) for differing time periods between 2010 and 2020 (see Table 1). Collars weighed 14 kg each, which was less than 1% of the weight of an adult elephant, the commonly accepted upper limit for radio collars^10^. For each elephant social unit, we collared only one individual. Males were solitary bulls except for one (Gading) who was often found traveling with various social units. All GPS units were set to record the location of the elephants at two-hour intervals, totalling 12 equally spaced GPS locations every day.

**Table 1:** Information on 14 collared elephants regarding their sex, start and end dates of collarings, the number of weeks with collared data, number of GPS points after data clean-up, and the year of land use spatial data used for the analysis.

Elephant	Sex	GPS collar start date	GPS collar end date	No. weeks collared	No. points in entire range	Spatial data used (year)
Aqeela	F	10/10/2010	06/12/2013	164.8	19,301	2010
Liun	F	08/01/2011	10/12/2013	152.5	9110	2010
Gading	M	25/10/2011	21/05/2012	37.9	1175	2010
*Recollar*		19/10/2012	17/12/2012			2010
Putut	F	26/10/2011	16/08/2012	42.1	847	2010
Jasmine	F	26/10/2011	13/05/2012	28.4	882	2010
Puteri	F	20/10/2012	07/12/2016	215.7	10,469	2015
Ita	F	02/06/2013	24/07/2014	59.4	4756	2015
Sejati	M	04/06/2013	30/10/2013	21.1	1749	2015
Sandi	F	19/06/2013	27/08/2015	114.1	9235	2015
Kasih	F	06/07/2014	20/06/2017	154.4	12,341	2015
Ratu	F	01/11/2016	18/02/2020	172.1	14,124	2015
Koyah	F	03/08/2016	06/10/2020	218	16,530	2015
Girang	F	20/08/2017	19/11/2019	117.2	18,289	2015
Sandy	M	31/10/2017	14/02/2020	119.4	8783	2015

To understand the extents of the entire known range for collared elephants, we mapped out all the data points at the individual level within ArcGIS 10.8.1. Using these data, we defined the extent of their range by creating a polygon (Minimum Convex Polygon) that linked the most exterior points of the dataset^51,52^. This was done in turn for each elephant, and once all 14 elephant ‘known’ ranges were mapped, these extents were pooled together in order to define the entire range used by these 14 individuals.

#### Mapping hot spots within elephant ranges

To better understand the areas most frequented by elephants (hot spots), we used a Moran’s I test to identify if, and to what degree, each collared dataset was clustered (i.e., whether spatial autocorrelation or clustering was present)^53,54^. Then, we undertook a hot spot analyses (Getis-Ord Gi*) to identify where spatial clustering occurred within the datasets^39–41^. Hot spot analyses calculates a z-score based on the Getis-Ord Gi* statistic that tells where features (in this case location points) have significantly high or low values that cluster spatially^55^. A larger z-score indicates a tendency towards more intense clustering of high values (hot spots) while a negative z-score indicates a tendency towards more intense clustering of low values (cold spots) (see Table SI 1 for z-score and corresponding p-value categories).

To undertake the Moran’s I and hot spot analyses, a number of steps were needed for each elephant dataset. First, GPS points within 100 m from another were ‘snapped’ together, using the Integrate tool in ArcGIS 10.8.1. Then, we pooled the snapped points, using the Collect Events tool in ArcGIS 10.8.1, to generate a new point layer that contained the count of the number of points aggregated within the 100 m distance. To test to see whether clustering was prevalent in each dataset (which is a prerequisite for hot spot analyses), we performed Moran’s I (Global) tool for spatial autocorrelation using the layers from the Collect Events tool. Next, we used the Hot Spot Analysis (Getis-Ord Gi*) tool, using the Collect Events output layers, and selected a fixed-distance band value of 2 km, which represented the average daily distance travelled by elephants in the Lower Kinabatangan floodplain^10^. The resulting output feature map displayed ‘hot spots’ and ‘cold spots’ along with respective confidence levels (Gi_Bin) and z-scores. We extracted the ‘hot spot’ points that had a 95% Confidence Interval or above (i.e., a z-score of 1.96 or above, see Table SI 1). To demarcate the extent of these hot spots we defined the extent of these points by creating a polygon that linked the most exterior points.

### Land use and land cover within elephant ranges and elephant hot spots

To understand land use and land cover within elephant ranges, we overlaid elephant ranges with land use/land cover data for 2010 or 2015, depending on when each elephant was collared (Table 1). For the 2010 land use/land cover dataset, we modified data from Abram et al.^47^. This 2010 data included detailed forest and oil palm spatial layers that defined forest types and age/productivity of oil palm areas^47^. For this analyses, however, we pooled all of the forest types into one generic forest area, and for the oil palm, we reclassified the extent into two classes, one consisting of oil palm within large industrial estates, and the other, oil palm in smallholdings. We demarcated these two classes based on the use of official land title information and distinctive visual cues (e.g. road patterns, planting regimes, size of planting blocks) seen in high resolution satellite imagery (SPOT 5 2.5 m) and Google Earth. For the oil palm smallholdings class, we expanded this layer by including village areas and dwellings as these land uses were often aggregated together. For the 2015 land use/land cover dataset, we used the 2010 layers and modified areas that had changed between the periods of 2010 to 2015, and used high resolution SPOT 5 1.5 m imagery to detect any changes in the landscape.

Classification accuracy was determined through the use of an error confusion matrix method and kappa statistics^56,57^. To do this, we used methods similar to Abram et al.^47^, and generated 230 random points (minimum 100 m apart) within ArcGIS 10.8.1 and imported these into Google Earth. Each point was assessed and assigned a class (either forest, oil palm estates, oil palm smallholdings/villages) for those points that covered high resolution tiles for their appropriate years (2010 and 2015). The assigned classes were then compared to the land use/land cover layers developed and an error confusion matrix table was used to assess data accuracy, and kappa statistics were calculated^56,57^.

We used government-provided spatial data to identify the protected areas within the region. These data included a number of protected areas and forest reserves under different designations including Wildlife Sanctuary (Sabah Wildlife Department), Class I Protection Forest Reserve, Class VI Virgin Jungle Reserve and Class VII Wildlife Reserve (Sabah Forestry Department), all named herein as ‘protected areas’ (Fig. 1). We used these data to then add an additional class to our land use and land cover spatial data for 2010 and 2015 that included: protected areas, unprotected forest, oil palm estates, and oil palm smallholdings/villages.

We also digitised the main roads in the landscape, using the SPOT 5 images to understand where these linear features are. To understand potential threats from other planned new roads/highways, we georeferenced and digitised relevant maps from the Sabah Structure Plan 2033, which is an overarching policy/planning document for the state^58^.

### Time spent analyses

We investigated the percentage of time that each collared elephant spent in their hot spots versus their entire range by calculating the proportion of GPS points (that represented time at 2 h intervals) within their hot spots. Further, we assessed the proportion of time spent for each collared elephant within our four land use/land cover classes, for their entire extent and for their hot spots. Additionally, for those elephants who had a hot spot that intersected with the existing or planned new roads/highway, we calculated time spent within those hot spots.

### Statistical analyses

We undertook four sets of statistical assessments of these data described above. First, we calculated Pearson correlation coefficients and conducted associated t-tests for independence to assess the pairwise (linear) relationships between the extent of an elephant’s entire range, the extent of the hot spot, the extent of the hot spot as a percentage of the entire extent, and the proportion of time spent in the hot spot.

Second, we undertook similar tests to evaluate the impact of the existing road and the planned road/highway. Specifically, we assessed correlations between an elephant’s: (a) entire extent and the proportion of time spent in hot spots that intersect the existing road and planned new road/highway; and, (b) proportion of time spent in hot spots and the proportion of time in hot spots that interested the existing road and planned new road/highway.

Third, to understand the variation between land use/land cover types within collared elephants entire ranges and hot spots, we conducted an analysis of variance (ANOVA) to assess whether the average proportion of extents for protected forest, unprotected forest, and oil palm estates were the same at the entire range, and hot spot levels. Only three land use types were used, as the use of oil palm smallholdings/villages were comparatively very small, and therefore somewhat obvious in regards to their significance. Further, if the four groups were included in these analyses then the proportions would add up to 1, which distorts the assumption of independent groups.

Fourth, we conducted a k-means cluster analysis to assess similarities and differences between elephants with respect to the proportion of time spent in the four types of land use/land cover across the entire range time, and in hot spots. Only values of k = 2 and 3 were considered, based on the sample size of 14 elephants. The choice of k was determined by comparing the between SS/total SS measure.

### Cadastral data and identifying land owners of oil palm estates

To enable us to understand the corporate stakeholders within elephant entire ranges and hot spots, we used available cadastral data that was ascertained from the Land and Survey Department’s online platform (http://www.jtuwma.net/) in January 2017. Through online searches for company information and reports, and by using the GeoRSPO online platform (https://rspo.org/members/georspo) we collated company level data on land title ownership, to the best of our ability, and incorporated this information into the cadastral (land title) layer. Additionally, we used the full extent of the cadastral layer, along with the extent of the protected areas data, to identify areas used by elephants that may be on state lands, meaning lands that have not yet been allocated (or alienated) under title, and therefore may be available for the government to easily include in their protected area network.

## Results

Collared elephant data ranged from 21.1 to 218 weeks for each individual (averaging 115.5 weeks), with nine out of 14 elephants having over 2 years of data, and four individuals having less than one year (Table 1).

The values of Moran’s I autocorrelation test (Table 2) showed that all elephant datasets z-scores were over 2.58 (meaning a less than 1% likelihood that clustering in the data is a result of random chance)^53,54^. In fact, for many individuals z-scores where very high, suggesting high presence of clustering^54^. This was also demonstrated with the very low *p* values, and Moran’s I values that ranged from 0.09 to 0.54, where 0 demotes perfect randomness^53^. As a result, all of the elephant datasets showed at least one or more statistically significant hot spot (Table 3).

**Table 2:** The summary of Moran’s I autocorrelation coefficient analysis for elephant collared data of 14 individuals. All *p* values were < 0.0001.

Elephant	Sex	Moran's I	z-score
Aqeela	F	0.26	25.5
Liun	F	0.17	24.8
Gading	M	0.18	29.2
Putut	F	0.091	4.6
Jasmine	F	0.12	5.7
Puteri	F	0.35	53.2
Ita	F	0.18	24.8
Sejati	M	0.12	21.8
Sandi	F	0.09	21.0
Kasih	F	0.15	64.0
Ratu	F	0.45	57.5
Koyah	F	0.54	60.9
Girang	F	0.30	48.8
Sandy	M	0.45	41.9

**Table 3:** Total extent for elephants entire range and hot spots (in km^2^), the proportion (%) of the extent of hot spots within the entire range, the number of hot spots and proportion (%) of time spent in hot spots.

Elephant	Sex	Extent for entire range (km^2^)	Extent for hot spots (km^2^)	Proportion (%) of hot spot extent within the entire range	No. of hot spots	Proportion (%) of time spent in hot spots
**Pooled Data**	**M/F**	**627.95**	**266.89**	**43%**	**9**	(*ave. 34%)
Aqeela	F	212.62	43.47	20%	4	38%
Liun	F	276.41	54.87	20%	6	35%
Gading	M	171.49	15.13	9%	1	33%
Putut	F	190.16	7.07	4%	3	10%
Jasmine	F	154.74	9.79	6%	2	15%
Puteri	F	418.43	53.54	13%	4	33%
Ita	F	247.11	19.91	8%	2	27%
Sejati	M	125.44	14.16	11%	2	60%
Sandi	F	259.10	32.89	13%	5	24%
Kasih	F	272.71	37.62	14%	5	35%
Ratu	F	375.70	72.53	19%	4	47%
Koyah	F	346.06	54.00	16%	3	43%
Girang	F	362.76	24.41	7%	6	20%
Sandy	M	221.42	28.76	13%	2	56%
**Average**		**263.37**	**33.44**	**12%**	**4**	**34%**

*The average calculated here is the average across the 14 elephants.

### Entire known ranges for the Kinabatangan elephants and relative importance of hot spots

Individually, entire known range extents averaged 263.37 km^2^, ranging from 125.44 to 418.43 km^2^; and for the hot spots, these ranged from one to six discrete areas (averaging four) and varied from 7.07 to 72.53 km^2^ in size (averaging 33.44 km^2^) (Table 3; Figs. SI 1 and SI 2). Correlation analysis revealed highly significant positive linear relationships between the extent of an elephant’s entire range and their respective hot spots (Table 3, r = 0.75, t = 3.97, *df* = 12, *p* = 0.0019), indicating that elephants with smaller entire range extents also have smaller sized hot spots.

Hot spots made up 4 to 20% (averaging 12%) of an individual’s entire range, however, time spent within these was high, ranging from 10 to 60% (averaging 34%, Table 3). The proportion of time spent in the hot spot was not associated with the absolute size of the entire range (r = 0.0023, *p* = 0.99) nor the absolute size of the hot spots (r = 0.39, *p* = 0.17). Instead, it was significantly positively associated with the relative size of hot spots (size of hot spot as a proportion of the entire range, r = 0.58, *p* = 0.031). Scatter plots indicated no alternative nonlinear or nonstandard relationships between the pairs of variables.

The pooled extent of all individuals entire ranges was 627.95 km^2^, which encompassed nine hot spots that spanned 266.89 km^2^ (or 43%) of the pooled entire known range extent (Table 3; Figs. 1, 2).

Nine, out of the 14, elephants had hot spots that intersected or boarded the existing main road and/or planned new road/highway. Hot spot extents ranged from 3.64 to 43.5 km^2^ (averaging 14.7 km^2^) (Table 4). For time spent in these hotspots, this ranged from 2 to 44% (averaging 14%) (Table 4). We found no significant correlation between an elephant's entire extent and the proportion of time spent by that elephant in hot spots intersecting roads (r = − 0.47, *p* = 0.20). However, there was a highly significant correlation between the proportion of time spent by an elephant in the hot spots and the proportion of time spent in hot spots intersecting roads.

**Table 4:** Occurrence of hot spot intersection with the main road or planned road/highway, along with the hot spot extent and proportion of time spent within that hot spot.

Elephant	Sex	Road intersection with entire range (ER) and hot spots (HS)	Extent (km^2^) of hot spot that intersects road	Proportion (%) of time spent in road intersected hot spot
Aqeela	F	ER & HS	43.47	26%
Liun	F	ER & HS	8.58	5%
Gading	M	ER	–	–
Putut	F	ER & HS	5.65	7%
Jasmine	F	ER	–	–
Puteri	F	ER & HS	3.64	2%
Ita	F	ER	–	–
Sejati	M	ER	–	–
Sandi	F	ER & HS	14.06	11%
Kasih	F	ER & HS	10.15	8%
Ratu	F	ER	–	–
Koyah	F	ER & HS	21.13	16%
Girang	F	ER & HS	7.75	8%
Sandy	M	ER & HS	18.02	44%

### Land use and land cover classification accuracy

Land use and land cover classification accuracy was 97.3%, with a Kappa statistic of 0.96 using an error matrix method and 226 points for 2010, and for 2015 the classification accuracy was 97.7%, with a Kappa statistic of 0.96 with 221 points (Tables SI 2 and 3). Both land use/land cover layers were deemed as good^56,59^.

### Extents of land use and land cover for entire ranges and hot spots

**Figure 3:**
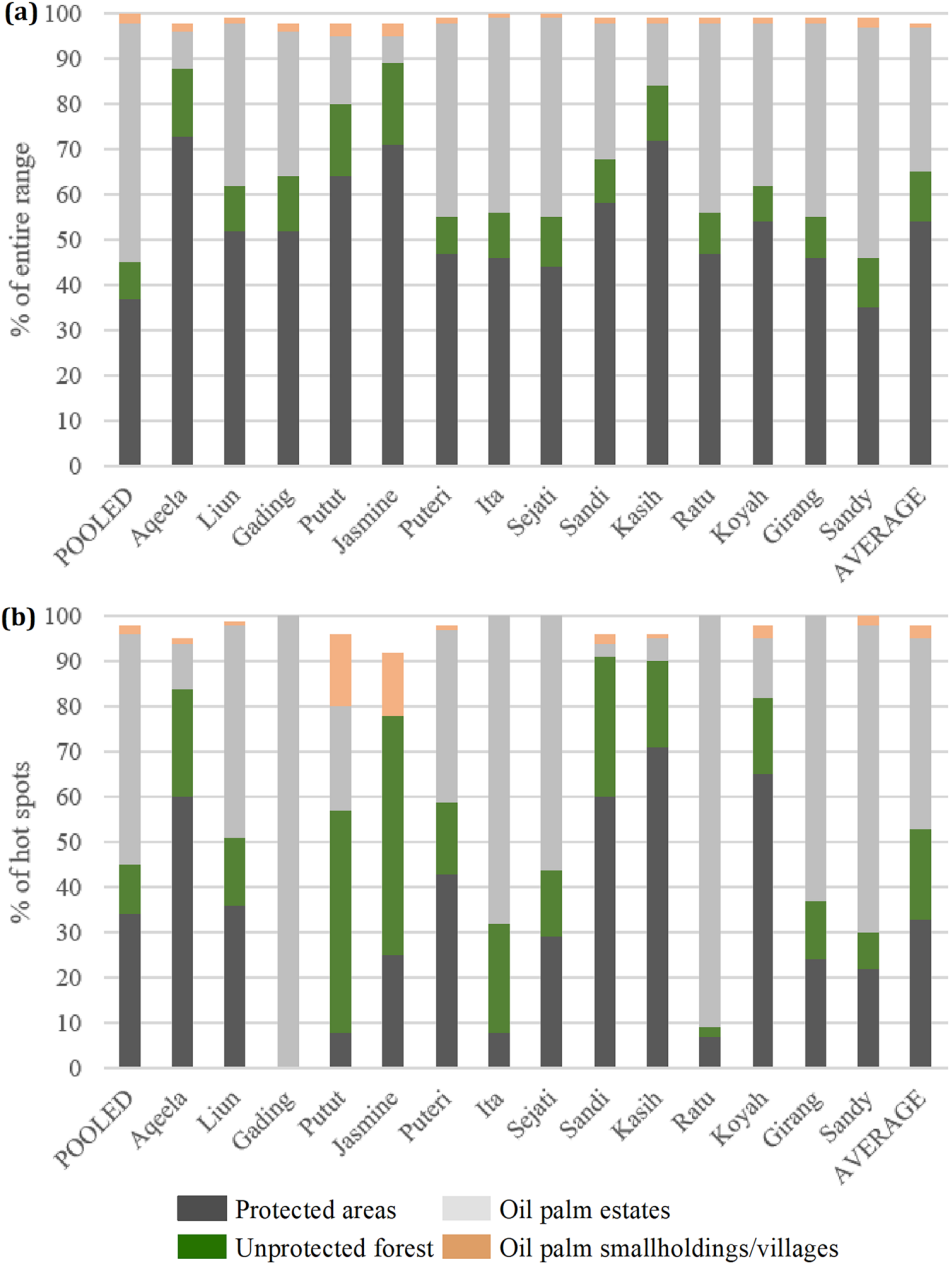
Proportions (%) of protected areas, areas of unprotected forest, areas of oil palm estates, and areas of village lands and oil palm smallholdings, within elephants (**a**) entire range, and (**b**) hot spots, along with pooled and average values.

At the individual level: forest habitats (protected and unprotected) were important and constituted 45 to 89% of elephants entire known range (averaging 66%); and from 0 to 91% (averaging 53%) within their hot spot extents (Fig. 3, Table SI 4). Oil palm estates ranged from 6 to 51% (averaging 32%) of the individuals entire range; and 0 to 100% (averaging 42%) within their hot spots (Fig. 3, Table SI 4). Extents of oil palm smallholdings/village areas were small across all elephant entire ranges and hot spots, typically ranging from 0 to 3%, except for two females who had this land use type as 14% and 16% of their hot spot extents (Fig. 3, Tables SI 4 and 5).

There was a highly significant difference between the average proportion of extents of protected areas, unprotected forest, and oil palm estates within elephants entire range (ANOVA F = 53; *df* = 2, 39; *p* = 7.6E−12). We also found an almost perfect negative linear association between the proportion of protected area extent versus oil palm estate extents across the entire range (correlation r = − 0.98, *p* = 1.8E−9). For hot spots, we found a substantive but not significant difference between the average proportion of extents of protected areas, unprotected forest, and oil palm estates (ANOVA F = 2.5; *df* = 2, 39; *p* = 0.094). The correlation between the proportion of protected area extent versus oil palm extent within the hot spots was also significantly negative (r = − 0.75, *p* = 0.0018).

For the pooled entire known range (627.95 km^2^), only 37% (231.35 km^2^) was in protected areas, 8% (49.09 km^2^) was in unprotected forests, 53% (331.08 km^2^) was in oil palm estates, and 2% (9.64 km^2^) was in oil palm smallholdings/villages (Fig. 3, Table SI 4). For the pooled hot spot extents, 34% (91.17 km^2^) was protected, 11% (29.12 km^2^) was in unprotected forests, 51% (136.88 km^2^) was in oil palm estates, and 2% (5.24 km^2^) was in oil palm smallholdings/village areas (Fig. 3, Table SI 4). Of the unprotected forests, at the entire range level (49.09 km^2^), 24.01 km^2^ was identified as potentially being on state land, with the remaining being on various land titles. For the unprotected forests identified in hot spots (29.12 km^2^), 15.83 km^2^ was identified as potentially being on state land.

### Time spent in land use and land cover for entire ranges and hot spots

Time spent in forest (protected and unprotected) ranged from 34 to 95% (averaging 65%) for their entire range; and from 0 to 98% (averaging 54%) within their hot spots (Table 5). For time spent in oil palm estates, this ranged from 0 to 65% (averaging 33%) for the entire known range, and from 0 to 100% (averaging 41%) in their hot spots; with six individuals spending the majority of their time in oil palm estates overall (Table 5).

**Table 5:** Percentage of time spent within four land use/land cover categories for the entire range and hot spots analyses, and cluster category from the k-means clustering.

Elephant	Proportion (%) of entire range time spent in:				Cluster for entire range	Proportion (%) of hot spot time spent in:				Cluster for hot spot
	Protected areas	Unprotected forest	Oil palm estates	Oil palm smallholdings/ villages		Protected areas	Unprotected forest	Oil palm estates	Oil palm smallholdings/ villages	
Aqeela	70%	26%	3%	0%	1	63%	36%	0%	1%	1
Liun	51%	16%	31%	1%	2	34%	19%	45%	0%	2
Gading	23%	11%	65%	1%	3	0%	0%	100%	0%	2
Putut	54%	29%	9%	6%	1	4%	42%	19%	33%	3
Jasmine	65%	30%	0%	4%	1	21%	61%	0%	18%	3
Puteri	54%	17%	28%	1%	2	41%	24%	34%	0%	1
Ita	34%	15%	49%	1%	3	1%	22%	76%	0%	2
Sejati	27%	14%	59%	1%	3	27%	14%	59%	0%	2
Sandi	63%	21%	13%	1%	1	51%	42%	4%	2%	1
Kasih	70%	22%	8%	0%	1	64%	33%	2%	0%	1
Ratu	30%	8%	61%	1%	3	5%	1%	94%	0%	2
Koyah	66%	14%	19%	0%	2	17%	18%	64%	1%	2
Girang	39%	10%	49%	1%	3	76%	18%	5%	0%	1
Sandy	22%	15%	61%	2%	3	16%	11%	73%	0%	2
Average	48%	18%	33%	1%		30%	24%	41%	4%	

The two k-means cluster analyses revealed three distinct groups of elephants with respect to the proportion of time they spent in the four land use/land cover types, across their entire extents and within their hot spots (Table 6). For both analyses, the between SS/total SS measures for k = 3 clusters (91.4%, 79.8%, respectively) were much higher than for k = 2 (83.4%, 64.9%).

**Table 6:** Number of elephants and proportions (%) of average time spent per cluster (derived from the k-means cluster analyses) for elephant’s entire range and hot spots.

	No. elephants in entire range clusters	Entire range				No. elephants in hot spot clusters	Hot spot			
		Protected areas	Unprotected forest	Oil palm estates	Oil palm smallholdings/villages		Protected areas	Unprotected forest	Oil palm estates	Oil palmsmallholdings/villages
Cluster 1	5	64%	26%	7%	2%	5	59%	31%	9%	> 1%
Cluster 2	3	57%	16%	26%	> 1%	7	14%	12%	73%	> 1%
Cluster 3	6	29%	12%	57%	> 1%	2	13%	52%	> 1%	25%

Across the entire range: five elephants were grouped into cluster 1, spending most of their time in protected areas (average 64%) and unprotected forest (26%), and very little time in oil palm estates (7%); three elephants were grouped into cluster 2 also spending a lot of time in protected areas (57%) and unprotected forest (16%) but also in oil palm estates (26%); and, six elephants were in cluster 3, spending over half their time (average 57%) in oil palm estates and much less time in protected areas (29%) and unprotected forest (12%) (Table 6). All three groups spent relatively little time on average in oil palm smallholdings/villages, although the elephants in cluster 1 spent the most time of all the groups in this catagory (Table 5).

Within the hot spots: seven individuals spent almost three-quarters of their time (73% on average) in oil palm estates; whereas elephants in the other two clusters spent around 10% of their time in this land use/land cover type. Five animals spent over half their time on average in protected areas (59%) and only a third of their time (31%) in unprotected forest, whereas the other cluster of two animals spent less time in protected areas (13%) and much more time in unprotected forest (52%) and oil palm smallholdings/villages (26%).

### Estate land owners in elephant ranges

**Figure 4:**
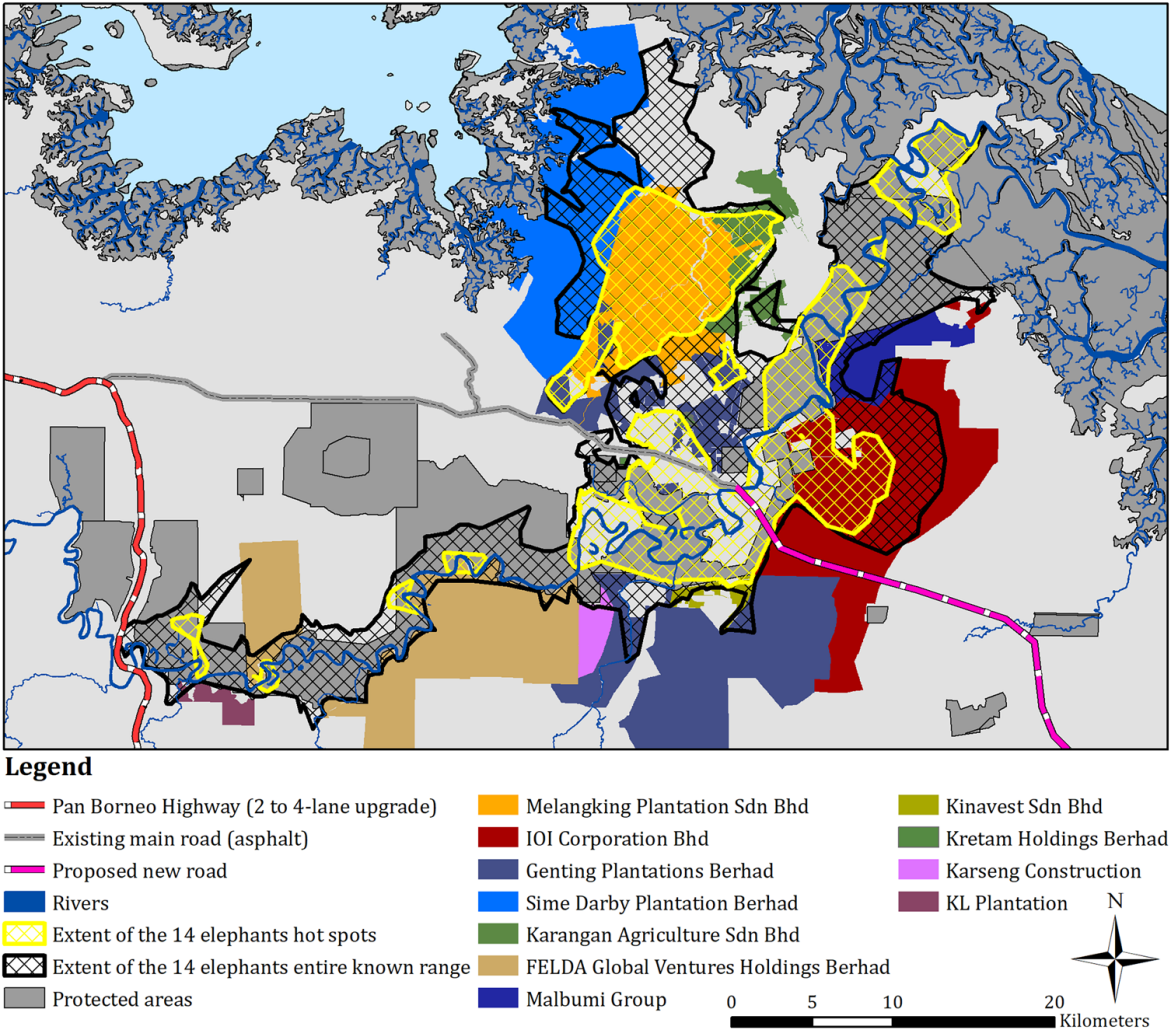
Map showing the pooled extents for hot spots (yellow cross hatch) and entire range (black cross hatch) within identified oil palm estates within the Lower Kinabatangan.

We identified eleven corporate land owners for 259.82 km^2^ (or 41%) of the 627.95 km^2^ pooled entire range, and nine corporate land owners for 115.84 km^2^ (or 43%) of the 266.89 km^2^ pooled hot spot extents (Table 7; Fig. 4). All of the identified estates had between 4 and 14 elephant entire ranges within their boundaries, and from 0 to 8 elephant hot spots (Table 7). Melangking Oil Palm Plantation had the greatest extent of both the pooled analyses within its estates (with 12 elephants using this estate within their entire range, and 6 within their hot spot extents), then IOI Corporation (with 11 elephants using this estate for their entire range and 8 elephants in their hot spots), Genting Plantations (with all elephants using this estate within their entire range and 7 in their hot spots), and Sime Darby (with 5 elephants using this estate in their entire range and 2 elephants in their hot spots), as well as a number of other palm oil companies (Table 7).

**Table 7:** Extents (km^2^) and proportions (%) of elephants entire range and hot spots within known oil palm estates, along with the companies RSPO membership status and number of collared elephants entire range and/or hot spots within estate boundaries. Table also shows extents within: unknown land tiles, protected areas, and in potential state lands.

Parent company/estate names	RSPO certified /member	Pooled entire range extent within oil palm estates	No. elephants entire range in estate	Pooled hot spot extent within oil palm estates	No. elephants hot spot in estate
Melangking Oil Palm Plantation		74.01 (12%)	12	63.30 (24%)	6
IOI Corporation	Yes	68.29 (11%)	11	28.01 (10%)	8
Genting Plantations	Yes	36.15 (6%)	14	9.02 (3%)	7
Sime Darby Plantation	Yes	35.92 (6%)	5	4.09 (2%)	2
Karangan Agriculture		16.86 (3%)	8	8.02 (3%)	2
FELDA Global Ventures Holdings	Yes	12.33 (2%)	13	0.69 (< 1%)	4
Malbumi Group		11.24 (2%)	13	2.22 (1%)	4
Kinavest		3.23 (1%)	13	0.30 (< 1%)	2
Kretam Holdings		0.76 (< 1%)	8	0.19 (< 1%)	1
Karseng Construction		0.73 (< 1%)	4	–	0
KL Plantation		0.30 (< 1%)	6	–	0
Areas with no information on titles		102.83 (16%)	–	38.56 14%)	–
Areas within protected areas		231.35 (37%)	–	91.17 (34%)	–
Potential areas of state lands		33.95 (5%)	–	21.32 (8%)	–
**Total extent**		**627.95 (100%)**	–	**266.89 (100%)**	–

There were 102.83 km^2^ (or 16%) of the pooled entire range, and 38.56 km^2^ (or 14%) of the pooled hot spot extents that had alienated titles (native titles and country lease titles) but ownership could not be identified. Further to this, 33.95 km^2^ (or 5%) of the pooled entire known range extent, and 21.32 km^2^ (8%) of the pooled hot spot extents could possibly be on state lands.

## Discussion

By pooling the results of the entire known range analysis of 14 GPS-collared elephants living in the Kinabatangan, our study suggests that this populations range covers at least 628 km^2^ (Table 3). Nine different locations were identified as hot spots, representing 266.9 km^2^ or 43% of this range, suggesting that just under half is highly used and/or frequented (Fig. 1). We found that the size of individual’s hot spots was positively related to the size of the entire range, meaning the larger the entire range the larger the summed area of an elephants hot spots. On average, hot spots represented a relatively small percent of an animal’s entire range (ranging from 4 to 20%, averaging 12%, Table 3). However, time spent within these hot spots ranged from 10 to 60% (averaging 34% across elephants, Table 5), with time spent in hot spots being related to the overall size of the hot spots (the larger the hot spot the more time elephants spent in them).

Identifying the location of these hot spots is essential in designing appropriate management practices in collaboration with land users and identifying the best location for elephant corridors. In the last 25 years, forest cover in the Lower Kinabatangan has been drastically reduced and fragmented^46^, eroding the biodiversity value of this landscape. Today, this region has little remaining forests, and what is left is insufficient for sustaining the local elephant population^10^. Moreover, forests are highly fragmented along the Kinabatangan River, with a number of bottlenecks constraining elephant movements^9^. The situation in this landscape is getting worse because of further land clearances for agriculture, namely oil palm; as well as for the highly controversial Sukau Bridge and new road/highway that is planned for the region.

Our analyses revealed a highly significant difference between the average proportions of protected area, unprotected forest, and oil palm estate extents within the elephant’s entire range; and a substantive, but not significant, difference across these land use/land cover types within hot spots (Table SI 4). At the individual level, there was a highly significant negative relationship between the proportion of protected areas and oil palm estates both within the elephant’s entire range and within the hot spots.

At the pooled level, we found that around 45% of the entire known range and hot spots were within forested environments (280.44 km^2^ and 120.29 km^2^ respectively). Our results showed strong fidelity of certain elephants to these forested habitats. Our k-means cluster analysis found that within elephant entire ranges and hot spots, two out of the three cluster groups had high or very high usage of forests. Both cluster 1, for the entire range, and cluster 1 for hot spots extents, had five females that on average used forest environments 90% of their time, with protected areas being used 64% and 59%, and unprotected forested being used on average 26% and 31%, respectively (Table 7).

Individuals in cluster 2, for the entire range analysis, on average, spent 73% of their time in forests (57% of this in protected areas and 16% in unprotected forests; Table 7). For the hot spot analysis, the individuals in cluster 2 spent on average 65% of their time in forests (52% of this in the unprotected forests and 13% in protected forests; Table 7). Elephants within these clusters were all females. Our results suggest that forest may be of particular importance for females as they had forest as their dominant land cover type within their entire range, hot spot extents and time spent analyses (Fig. 3, Table 5). Several studies have shown that adult females influence and guide the movement patterns and habitat utilization for their family group and that females in family units tend to inhabit less risky areas, such as within natural forest habitat^60–62^.

However, the unprotected forest is at risk. We identified about 8% (or 49 km^2^) of forest identified within the pooled entire known range were not protected, with half potentially being on state land, and the remaining half on land titles of various types (Table SI 4). For the pooled hot spot areas, unprotected forest was proportionally higher, comprising of 11% (or 29 km^2^) of the total extent, with 54% being potentially on State land and 46% on land titles (Table SI 4). Protecting these forests would be an essential and efficient way to secure key elephant habitat since all collared individuals were using these forest fragments in their entire range (averaging 11%, and ranging from 8 to 18%), and hot spot extents (averaging 20%, and ranging from 0 to 53%) (Table SI 4, Fig. 3). On average, 24% of time was spent in unprotected forests within hot spots, though this varied widely from 0% (for the male elephant known as Gading) to 61% (for the female matriarch named Jasmine) (Table 5). In fact, five females had large proportions of their hot spot extents (24–53%) in unprotected forests, spending substantial periods of their time (33–61%) within these threatened areas.

Our findings show that unprotected forests around the villages of Bilit and Sukau, were of particular significance (Figs. 1, 2). These unprotected forests largely consist of lowland dry forest, seasonally flooded swamp forest, and swamp forest, which are considered important habitats for elephants for feeding, resting and moving^47,63^. Within these forests, and along the forest margins and river banks there are also natural open grasslands that consist of *Phragmites karka* and *Dinochloa scabrida* that provide essential forage, mainly in the riparian areas for elephants^9,21,23^. Forested environments are also considered to be important in providing natural refugee from human activities and disturbance. For example, elephants have been documented to form significantly larger group sizes, as well as engaging in significantly more social interactions, in natural forest habitat compared to, for example, oil palm landscapes^63^. Adult females, generally, avoid areas considered unsafe for their respective social units, are more selective in the resources they use, and require regular access to water because of the presence of young^64–66^. This may be why our results, strongly suggest that forest habitats seem to be most important for adult females.

Another significant issue faced by these elephants is the threat from the controversial planned Sukau bridge and road/highway that is set out in the Sabah Structure Plan, an overarching policy document for the State^58^. Currently, a new road/highway is under construction on the northern bank of the village of Sukau, and this has already cleared areas of unprotected forest. This public road could link to a potential new bridge that would cross over the Kinabatangan River, cutting through unprotected forest and a protected area (Lower Kinabatangan Wildlife Sanctuary), before going through oil palm estates then through another protected area to the south and through the Tabin elephant population range. For the Kinabatangan, creating a public highway will cut the elephant population range into two parts (Figs. 2, 3). All collared elephants use this area, as it is a key bottleneck and the only alternative option to pass around Sukau village^9^. We found that nine elephants have hot spots that intersect or meet up with the current road (which will be up-graded and get considerably busier) and/or the planned road/highway alignment (Figs. SI 1 and 2). For these elephants, we calculated that they spent from 2 to 44% (average 14%) of their time within these hot spots (Table 4). Our statistical analyses suggest that if the road/highway goes ahead it will have a significant impact on the elephants’ behaviour with respect to time spent in the hot spots. Indeed, this infrastructure project could have dire consequences for these elephants and their family groups, by disrupting their ranging patterns and segmenting the entire elephant range into two (Figs. 2, 4). The existing road in Batu Putih has already proven to be an impassable barrier for this elephant population, as no elephants have been observed crossing this road since the early 2000s^14^. For elephants that do try and cross, vehicle collisions may become a significant threat to elephants and drivers alike^67^, and potentially increasing human–elephant conflict in the nearby villages, as well as in plantations^14,68,69^, exacerbating an already difficult situation for this small and fragmented population.

Results from the pooled analysis show that about 53% of the entire known population range is within oil palm estates; and 51% for the pooled hot spots (Fig. 3, Table SI 4). Our k-means clustering analysis grouped 6 elephants into cluster 3 that on average spent 57% of time in oil palm estates; and 7 elephants into cluster 2 within the hot spot analysis that on average spent 73% of their time in oil palm estates (Table 6). All the males, were clustered within these groups (Table 5). In fact, the three collared males were amongst the highest users of oil palm estates (Fig. 3, Table SI4, and 5). This could be related to a ‘‘high risk, high gain’’ strategy, often adopted by males to increase body size and enhance reproductive success^32,33,60^. However, it is interesting to see that three females (Ita, Ratu and Koyah) and their respective social units, also seemed to have high levels of oil palm use, while other individuals had zero or very little use of oil palm (e.g. Aqeela, Jasmin, Sandi, Kasih; Table SI 4, Fig. 3). Differential choices may result from differences in individual knowledge and experience with people during past encounters, for example^70,71^.

We identified that collared elephants were ranging in 11 known oil palm estates, with the five most regularly used being Melangking Oil Palm Plantation (with 12 elephants entire range overlapping with this estate and six hot spots), IOI Corporation (with 11 overlapping entire ranges, and eight hot spots), Genting Plantations (14 and seven, respectively), Sime Darby Plantation (five and two, respectively), and Karangan Agriculture (8 and 2, respectively) (Table 6; Fig. 4). Presence of bottlenecks and barriers (e.g. electric fences) may explain hot spot occurrences in these estates, as well as feeding opportunities, management strategies of specific estates, and historical and seasonal ranges.

Linear features like major highways, electric fences and drainage ditches hamper elephant movements within the Lower Kinabatangan^9^. A previous study identified 20 bottlenecks in the Lower Kinabatangan with the two main ones (of 9 km and 6.5 km in length) found around the village of Sukau^9^. In addition, the unplanned and chaotic erection of electric fences by large estates and smallholdings has disrupted significantly elephant movement patterns and resulted in artificial hot spots for certain individuals (e.g. Liun, Ita, Gading and Sejati)^35,72^. Electric fences have widely been used to mitigate human–elephant conflicts. The establishment of fences rarely consider the traditional elephant routes nor the location of existing fences in neighbouring estates. If elephants manage to enter such areas, they often become trapped and experience difficulties in returning to nearby forests, exacerbating conflicts with people^35^.

Certain estates such as Melangking Oil Palm Plantation have allowed elephants to roam freely in their estate (Muhammad Al-Shafieq, personal communication). Since 2017, this plantation has shown a drastic reduction in damages to their oil palms following the removal of their permanent electric fences surrounding their entire estate. Instead, this plantation is using a temporary electric fencing regime around newly planted palm areas. Concurrently, they now do not push elephants out of their estate, which can explain why Melangking Oil Palm Plantation is a significant hotspot in the region.

Another reason why elephant ranges incorporate oil palm estates is to move between forest patches that are becoming completely isolated following forest conversion, as is the case close to Sukau (Fig. SI1 and SI2; Fig. 1). Unlike other elephant species that increase their speed of movement rates in highly disturbed areas^27,30,66^, the Bornean elephant has been observed doing the opposite, which may explain some of the hot spots within oil palm estates. This movement strategy may allow for better vigilance as seen on a few occasions when elephants spent 2–5 days in the Bukit Melapi-Yu Kwang Corridor, near the village of Sukau, before leaving the area (Othman, personal observation).

Hot spots in the oil palm landscape can also be explained by feeding opportunities, since elephants feed on palm shoots, leaves and hearts^73^. Elephants are known to eat the shoots of newly planted oil palms, often killing the palms and causing significant economic damages^35^. Since 2010, many estates located in the Lower Kinabatangan have started a new palm rotation. Palms are replanted every 25 years. A new rotation includes land clearing, bole and root mass removal, and the shredding or chipping of felled palms. Elephants are attracted to the shredded palm hearts since it gives them easy access to one of their favourite food^72^. This particular behaviour does not cause economic damage, and some estate managers allow the elephants to stay and forage in the chipping areas. This was documented for several collared elephants, whose hot spots and time spent were particularly high within oil palm (e.g. Gading and Sandy, two males; and Ratu and Ita, two females). Once the shredded palms have dried, however, elephants will leave these areas and move elsewhere. Within oil palm estates, some elephants have been found to travel more directly and rapidly suggesting ‘exploratory’ behaviour, which could be associated with searching for young palms or areas of palm felling and chipping of palm hearts^15^.

Lastly, elephants may still be using their historical range that used to be covered with forest before conversion to oil palm. Other factors potentially explaining the relatively high use of oil palm estates include seasonal variations of ranging patterns. Indeed events of drought or floods limit the access to various parts of the floodplain and will tend to confine the animals in some areas^9,63^.

In Sabah the state authorities have recorded at least 200 elephant deaths from the year 2010 to 2021 and most of these have occurred on, or near, oil palm estates^14,74–76^. Deaths from non-natural causes are largely due to poisoning (both accidental and intentional), gunshot wounds, poaching for tusks and other body parts, and snares^35^. Stopping killing and enabling a safe coexistence between people and elephants within multiple-use landscapes that are dominated by oil palm is one of the key strategies developed in the Bornean Elephant Action Plan for Sabah (2020–2029), which was endorsed by the State^14^. Based on our results in Lower Kinabatangan, a series of recommendations are proposed.

This study underscores the importance of remaining forested areas for the Lower Kinabatangan elephant population. Full protection of all forest fragments left in the Lower Kinabatangan is urgently needed. Several official mechanisms are available to fulfil this request that has been proposed for the past 20 years by various organizations^46^.

The current network of forests available in the Lower Kinabatangan is too small and fragmented to sustain a viable elephant population. Forest corridors must be created across the landscape through reforestation exercises, whilst concurrently undertaking enrichment planting of native understory forage within forested areas as this may minimize the need for elephants to search for easily accessible food in high-risk oil palm landscapes^21–23^.

Current governmental plans to build a road bridge and public road/highway linking the southern bank of the Kinabatangan River to Tabin Wildlife Reserve to the south will irreversibly impact the Lower Kinabatangan elephant population by cutting the current range into two isolated parts. This will impact the elephants ranging patterns, potentially even fragmenting the already small population into two groups, and potentially leading to elephant deaths by vehicle collisions (which is becoming increasingly common in Peninsular Malaysia), and increase the risk of poaching activities, all resulting in a decrease in the genetic diversity of the, already small and isolated, population^14,67^.

Eventually, the future of the Kinabatangan elephant population resides in improving land use and management practices within oil palm estates currently used by elephants. We recommend that priority should be given at improving elephant movements in oil palm estates by removing unnecessary man-made barriers and only cautiously installing temporary electric fences to protect sensitive areas. For example, the use of electric fences around mature oil palm and areas whereby palms are being removed and chipped could be prohibited, and electric fences permitted solely for protecting oil palm nurseries, new plantings and young oil palms (e.g. up to 7–8 years old), and staff and office quarters. This would greatly allow for landscape permeability for elephants, and other species that need to cross the landscape for their ecological and biological needs^14^.

A handful of guidelines exist to assist oil palm managers and staff in managing elephant populations in their respective estates^72,77^. However, there is a need for a more comprehensive set of guidelines, which delineate better practices with the aim to increase the protection of people and elephants outside protected areas. Guidelines should specify “do’s” and “don’ts” (based on best available data and knowledge) of actions needed before, during and after elephants visit oil palm estates and smallholdings.

Sabah now is in an interesting transition within their palm oil sector. On the 21st October 2015, the Sabah State Cabinet committed to produce 100% certified sustainable palm oil, by 2025, under the Roundtable for Sustainable palm Oil (RSPO) Jurisdictional Certification approach. Under this approach, areas of High Conservation Value and areas identified within the High Carbon Stock Approach need specific management and monitoring, in order to comply with RSPO principles and criteria^78–80^. Sabah government can use this platform to build an integrated landscape level approach to better manage landscapes within known elephant ranges (which is considered a High Conservation Value species) to allow for a safe and permeable movement through the landscape.

Eventually, long-term survival of the Bornean elephant will mainly depend on how people and elephants can co-exist. It is our hope that this study illustrates the importance of protecting all forested habitat and effectively managing areas outside of protected areas to allow for long-term elephant coexistence with humans in this landscape.

## Supplementary Information

Supplementary Information.

## Data Availability

The datasets analyzed during the current study are not publicly available due to the potential misuse of those data to locate an endangered species threatened by poaching, but the datasets are available from the corresponding authors on reasonable request.
