# Supplementary material for: Understanding the spatial distribution and hot spots of collared Bornean elephants in a multi-use landscape
Source: Sci Rep. 2022 Jul 27;12:12830. doi: 10.1038/s41598-022-16630-4 (PMC9329282; doi:10.1038/s41598-022-16630-4)
Supplement: Supplementary file 1 — Supplementary Information. [file 41598_2022_16630_MOESM1_ESM.pdf]

Supporting Information

Understanding the spatial distribution and hot spots of collared Bornean elephants in a multi-use landscape

Abram, N.K.[1*,] Skara, B.[2,], Othman, N.[3,4] Ancrenaz, M.[5], Mengersen, K.[6], Goossens, B.[7,8,9,10*]

[1]Forever Sabah, H30 Gaya Park, Lorong Muntahan 1C, Penampang Road, 88300 Kota Kinabalu, Sabah, Malaysia

[2]Institute for Biodiversity and Ecosystem Dynamics, University of Amsterdam, Science Park 904, 1098 XH Amsterdam, Netherlands

[3]Sabah Biodiversity Conservation Association (Seratu Aatai), S10, 1st Floor, Block B, Peak Vista 88400 Kota Kinabalu, Sabah

[4] Institute for Tropical Biology and Conservation, Universiti Malaysia Sabah, 88400 Kota Kinabalu, Sabah, Malaysia

[5]HUTAN/Kinabatangan Orang-utan Conservation Programme, 88874 Kota Kinabalu, Sabah, Malaysia

[6]Centre for Data Science and School of Mathematical Sciences, Queensland University of Technology, 2 George St, Brisbane 4001, Queensland, Australia

[7]Danau Girang Field Centre, c/o Sabah Wildlife Department, Wisma Muis, 88100 Kota Kinabalu, Sabah, Malaysia

[8]Organisms and Environment Division, Cardiff School of Biosciences, Cardiff University, Sir Martin Evans Building, Museum Avenue, Cardiff CF10 3AX, UK

[9]Sustainable Places Research Institute, Cardiff University, 33 Park Place, Cardiff CF10 3BA, UK

[10]Sabah Wildlife Department, Wisma Muis, 88100 Kota Kinabalu, Sabah, Malaysia

Corresponding authors: Nicola Karen Abram (nicola_abram@hotmail.com) and Benoit Goossens (goossensbr@cardiff.ac.uk)

Table SI 1. Summary of the categories of z-scores and corresponding *p*-values and Confidence Intervals for Moran's I and hot spot analyses. The z-score measures degree of clustering with scores near 0 indicating no significant clustering. Z-scores have corresponding *p*-values. A *p*-values of 0.05 (or less) with high positive z-scores indicate a clustering of significantly high values (hot spot), and p-value of 0.05 (or less) with high negative z-values indicate a clustering of significantly low values (cold spot).

Critical Value (z-score)	Significance level (p-value)	Confidence Interval
<-2.58	0.01	99% Confidence
-2.58 - 1.96	0.05	95% Confidence
-1.96 - 1.65	0.10	90% Confidence
-1.65 - 1.65	Not significant	Not significant
1.65 - 1.96	0.10	90% Confidence
1.96 - 2.58	0.05	95% Confidence
>2.58	0.01	99% Confidence

Table SI 2. Confusion matrix table of Land Use and Land Cover (LULC) for 2010. The overall accuracy of the LULC was 97.3%, based on 226 points. Oil palm estates and oil smallholdings/villages were perfectly classified. Forest had an accuracy of 94%.

**LULC 2010**	**Forest**	**Oil palm estate**	**Smallholdings/villages**	**Total**
**Forest**	96	0	0	96
**Oil palm estate**	3	97	0	100
**Oil Palm smallholdings/villages**	3	0	27	30
**Total**	102	97	27	226
**Correctly Classified %**	94%	100%	100%	

Table SI 3. Confusion matrix table of Land Use and Land Cover (LULC) for 2015. The overall accuracy of the LULC was 97.7%, based on 221 points. Oil palm estates and oil smallholdings/villages were perfectly classified. Forest had an accuracy of 93%.

**LULC 2015**	**Forest**	**Oil palm estate**	**Smallholdings/villages**	**Total**
**Forest**	68	0	0	68
**Oil palm estate**	0	119	0	119
**Oil Palm smallholdings/villages**	5	0	29	34
**Total**	73	119	29	221
**Correctly Classified %**	93%	100%	100%	

Table SI 4. Extents (km^2) and proportions (%) of the entire range and hot spot extents in protected areas, unprotected forest, oil palm estates, and oil palm smallholdings/villages.

		Extents (km^2) and proportions (%) of entire range in:				Extents (km^2) and proportions (%) of hot spots in:			
**Elephant**	**Sex**	**Protected areas**	**Unprotected forest**	**Oil palm estates**	**Oil palm small-holdings/ villages**	**Protected areas**	**Unprotected forest**	**Oil palm estates**	**Oil palm small-holdings/ villages**
Pooled data	F/M	231.35 (37%)	49.09 (8%)	331.08 (53%)	9.64 (2%)	91.17 (34%)	29.12 (11%)	136.88 (51%)	5.24 (2%)
Aqeela	F	156.24 (73%)	32.23 (15%)	16.52 (8%)	3.88 (2%)	37.75 (60%)	15.08 (24%)	6.45 (10%)	0.71 (1%)
Liun	F	142.54 (52%)	28.94 (10%)	99.82 (36%)	1.90 (1%)	18.13 (36%)	7.50 (15%)	23.89 (47%)	0.36 (1%)
Gading	M	94.20 (52%)	21.40 (12%)	58.69 (32%)	3.88 (2%)	0 (0%)	0 (0%)	15.13 (100%)	0 (0%)
Putut	F	130.79 (64%)	33.66 (16%)	30.01 (15%)	5.77 (3%)	0.60 (8%)	3.61 (49%)	1.72 (23%)	1.18 (16%)
Jasmine	F	119.15 (71%)	29.50 (18%)	10.75 (6%)	4.69 (3%)	2.80 (25%)	5.95 (53%)	0 (0%)	1.55 (14%)
Puteri	F	196.60 (47%)	34.45 (8%)	180.10 (43%)	3.24 (1%)	21.54 (43%)	8.26 (16%)	19.36 (38%)	0.51 (1%)
Ita	F	113.57 (46%)	24.75 (10%)	106.48 (43%)	2.31 (1%)	1.64 (8%)	4.75 (24%)	13.50 (68%)	0.03 (0%)
Sejati	M	55.37 (44%)	13.35 (11%)	55.68 (44%)	1.04 (1%)	4.04 (29%)	2.11 (15%)	8.01 (57%)	0 (0%)
Sandi	F	159.10 (58%)	27.77 (10%)	83.04 (30%)	2.58 (1%)	19.64 (60%)	10.09 (31%)	1.01 (3%)	0.80 (2%)
Kasih	F	196.28 (72%)	32.21 (12%)	37.68 (14%)	2.88 (1%)	26.85 (71%)	7.25 (19%)	1.94 (5%)	0.45 (1%)
Ratu	F	178.30 (47%)	32.52 (9%)	158.35 (42%)	2.42 (1%)	5.26 (7%)	1.35 (2%)	65.83 (91%)	0 (0%)
Koyah	F	187.23 (54%)	29.15 (8%)	123.56 (36%)	2.24 (1%)	35.37 (65%)	9.32 (17%)	6.95 (13%)	1.46 (3%)
Girang	F	168.33 (46%)	31.51 (9%)	155.46 (43%)	2.92 (1%)	5.97 (24%)	3.12 (13%)	15.27 (63%)	0 (0%)
Sandy	M	76.54 (35%)	24.14 (11%)	113.91 (51%)	3.90 (2%)	6.23 (22%)	2.17 (8%)	19.55 (68%)	0.80 (3%)
Average		147.04 (54%)	29.64 (11%)	104.08 (32%)	3.565 (1%)	18.47 (33%)	7.31 (20%)	22.37 (42%)	0.87 (3%)

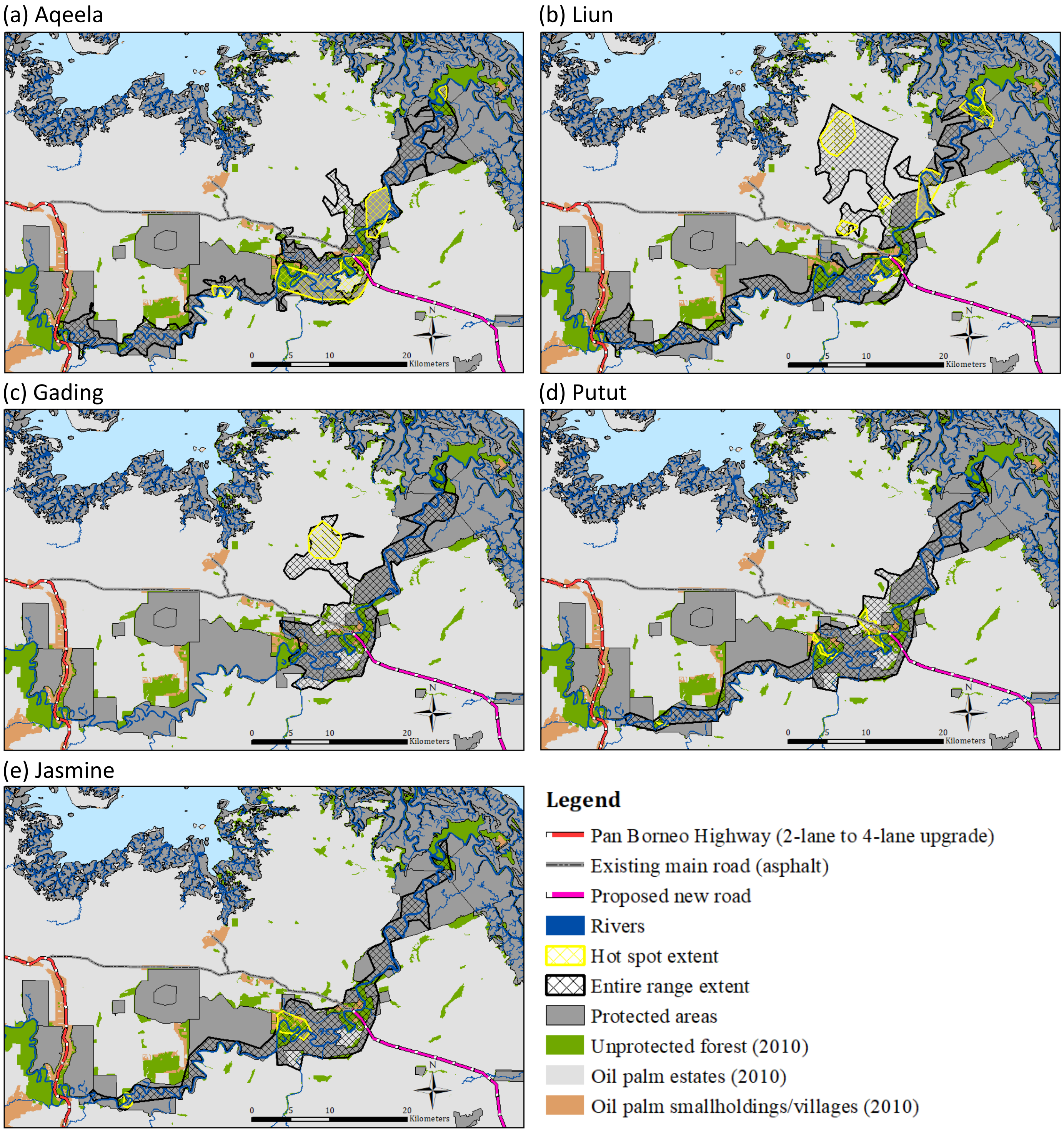

Figure SI 1. Maps showing the hot spot areas (yellow cross hatch) inside of the mapped out entire range (black cross hatch) for each collared elephant in the Lower Kinabatangan; along with information on locations of protected areas, unprotected forest, oil palm estates, and oil palm smallholdings/villages in 2010.

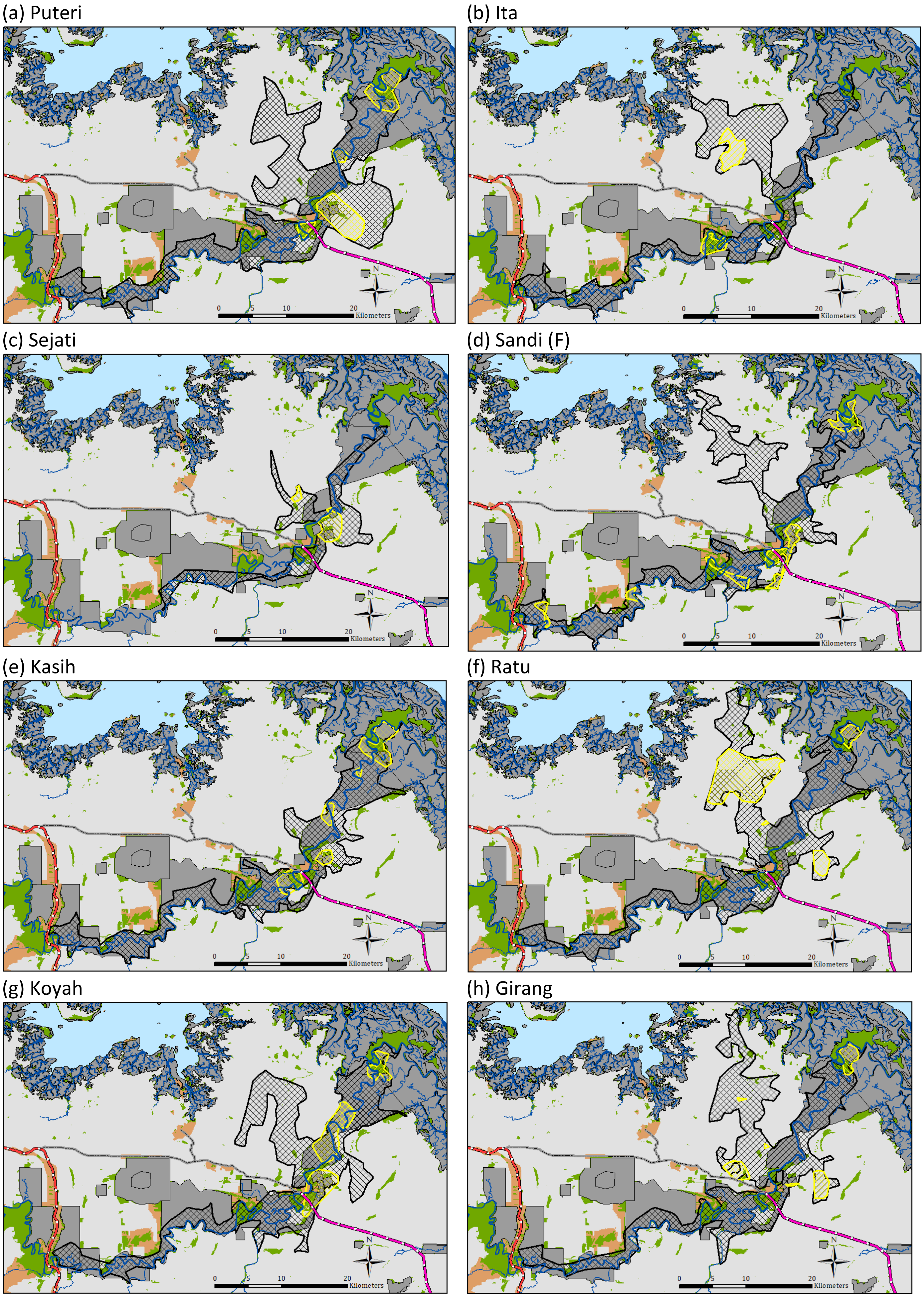
(a) Puteri
(b) Ita
(c) Sejati
(d) Sandi (F)
(e) Kasih
(f) Ratu
(g) Koyah
(h) Girang
N
0
5
10
20
Kilometers

(i) Sandy (M)

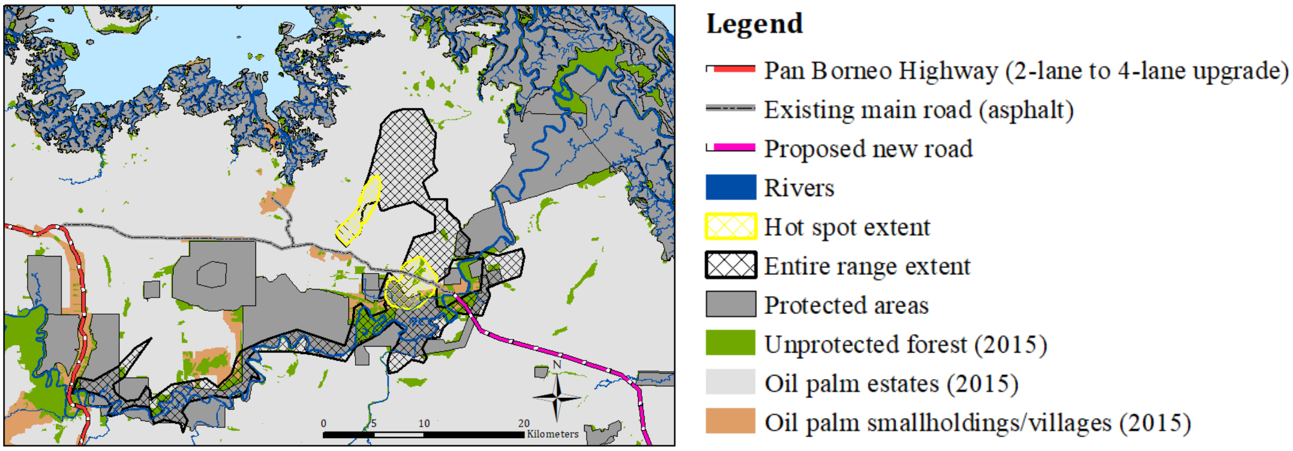

Figure SI 2. Maps showing the hot spot areas (yellow cross hatch) inside of the mapped out entire range (black cross hatch) for each collared elephant in the Lower Kinabatangan; along with information on locations of protected areas, unprotected forest, oil palm estates, and oil palm smallholdings/villages in 2015.
